# Cluster randomized controlled trial of a psycho-educational intervention for people with a family history of depression for use in general practice

**DOI:** 10.1186/1471-244X-13-325

**Published:** 2013-12-01

**Authors:** Bettina Meiser, Peter R Schofield, Lyndal Trevena, Alex Wilde, Kristine Barlow-Stewart, Judy Proudfoot, Michelle Peate, Timothy Dobbins, Helen Christensen, Kerry A Sherman, Janan Karatas, Philip B Mitchell

**Affiliations:** 1 Psychosocial Research Group, Prince of Wales Clinical School, The University of New Sourth Wales, Sydney, NSW 2052, Australia; 2 Neuroscience Research Australia, Sydney, NSW 2031, Australia; 3 School of Medical Sciences, University of New South Wales, Sydney, NSW 2052, Australia; 4 Sydney School of Public Health, University of Sydney, Sydney, NSW 2006, Australia; 5 School of Psychiatry, University of New South Wales, Sydney, NSW 2052, Australia; 6 Black Dog Institute, Sydney, NSW 2031, Australia; 7 Centre for Emotional Health, Department of Psychology, Macquarie University, Sydney, NSW 2109, Australia; 8 Centre for Genetics Education, NSW Health, Royal North Shore Hospital, St Leonards, NSW 2065, Australia

**Keywords:** Family history, Major depressive disorder, Bipolar disorder, Online intervention, Psycho-education, Prevention

## Abstract

**Background:**

The strongest risk factor for depression is having a family history of the condition. Many individuals with a family history of depression are concerned about their personal risk for depression and report unmet educational and psychological support needs. No supportive and/or educational interventions are currently available that target this group of individuals. In this study we will develop and evaluate the first online psycho-educational intervention targeted to individuals with a family history of depression. Genetic risk information and evidence-rated information on preventive strategies for depression will be provided to such individuals in a general practice setting. The intervention will also incorporate a risk assessment tool. The content and delivery of the intervention will be pilot-tested.

**Methods/design:**

The proposed intervention will be evaluated in the general practitioner (GPs) setting, using a cluster randomized controlled trial. GP practices will be randomized to provide either access to the online, targeted psycho-educational intervention or brief generic information about depression (control) to eligible patients. Eligibility criteria include having at least one first-degree relative with either major depressive disorder (MDD) or bipolar disorder (BD). The primary outcome measure is 'intention to adopt, or actual adoption of, risk-reducing strategies’. Secondary outcome measures include: depression symptoms, perceived stigma of depression, knowledge of risk factors for development of depression and risk-reducing strategies, and perceived risk of developing depression or having a recurrence of family history. Over the course of the study, participants will complete online questionnaires at three time points: at baseline, and two weeks and six months after receiving the intervention or control condition.

**Discussion:**

This novel psycho-educational intervention will provide individuals with a family history of depression with information on evidence-based strategies for the prevention of depression, thus, we hypothesize, enabling them to make appropriate lifestyle choices and implement behaviors designed to reduce their risk for depression. The online psycho-educational intervention will also provide a model for similar interventions aimed at individuals at increased familial risk for other psychiatric disorders.

**Trial registration:**

The study is registered with the Australian and New Zealand Clinical Trials Group (Registration no: ACTRN12613000402741).

## Background

Having a family history of depression is the strongest documented risk factor for depression [1]. Family, twin, and adoption studies strongly suggest that both major depressive disorder (MDD) and bipolar disorder (BD) have heritable components [2], with heritability estimates of 40% for MDD [1,3] and 80% for BD [4,5].

A meta-analysis of family studies of MDD found a relative risk of 2.8 for MDD in first-degree relatives of affected probands, compared to control subjects [1], which is greater than all other reported risk factors. The presence of multiple relatives with MDD, as well as early age of onset, recurrence and severity of the depression in affected relatives, are factors that indicate an even higher familial risk [6]. The magnitude of familial aggregation for BD is extremely high; the relative risk in first-degree relatives of probands with BD compared to control cases has been estimated at 13.6 [7].

Genes that are responsible for an inherited susceptibility to depression are still the focus of intensive research efforts; genetic testing for susceptibility to mental health conditions is not available for clinical use. Given this, family history is currently the best predictor for the development of MDD and BD [6]. The role of family history as a key risk factor is undisputed, and epidemiological data can be used to provide people with a family history with estimates of their risk.

Many individuals with a family history of depression report unmet educational and psychological support needs in relation to a range of issues, including: estimating their personal risk of developing depression, as well as the risk to offspring; learning how depression can be prevented in those at increased risk based on family history, as well as early intervention and risk modification in young relatives; how to cope with family vulnerability; and reproductive decision-making [8,9]. Despite this well-documented endorsement of the need for psycho-educational interventions, no such interventions are currently available targeting this group of individuals.

Virtually nothing is known about the optimal approaches to providing risk information to people with a family history of depression. Professional guidelines suggest that genetic risk information should be offered to people with BD and their families, particularly those who are considering having children [10]. The following groups of individuals may benefit from risk information in particular: adult children, siblings and parents of affected individuals; affected individuals and their partners planning their families; and, affected people who maintain high-risk behaviors [11]. Indeed, as many as 75% of individuals with BD report that they would like expert advice about their genetic risk [12]. The guidelines, however, do not provide guidance on how to deliver the information.

Despite the potential value of information provision about familial risk, surveys of genetic counselors and psychiatrists show that they feel ill equipped to provide patients with such information [13-15]. Estimating the risks for the development of depression is challenging, given the lack of systematic reviews and meta-analyses available on more complex family relationships (e.g. risk associated with having a second-degree rather than first-degree relative with depression, presence of multiple affected relatives on either one side only or both sides of the family), and possible presence of a heterogeneous group of disorders within a given family.

Finally, further complexity is introduced through the contribution of environmental factors, which may be either familial or non-familial. Environmental familial factors pertain to the shared familial environment (e.g. parenting style), and environmental non-familial factors relate to the known contribution of sociodemographic variables, individual environmental (e.g. stressful life events) and health-related factors (e.g. presence of non-psychiatric chronic illness). It should, however, be noted that empirical risks reported by family studies (as distinct from adoption and twin studies) do not allow for a differentiation between familial genetic and environmental contributors to depression risk [1].

It is critical that information on familial risk for depression to individuals at increased risk is accompanied by the provision of advice on early detection, risk management and prevention. For example, individuals at increased familial risk should be advised to undergo regular screening by a health care provider in a timely manner, to capitalize on the predictive power of early warning signs and intervene preventively [16], in accordance with new prevention frameworks in psychiatry, which regard minor depression as a risk factor for MDD [17]. While several risk factors for depression, including family history, cannot be changed, individuals at increased risk are likely to benefit from accurate and up-to-date information on risk factors that are amenable to change and/or strategies they may adopt to reduce their risk. Such risk management strategies may either be based on evidence from randomized controlled trails (RCTs) (e.g. cognitive behavioral therapy [CBT], regular exercise) [18-22] or represent potential risk-reducing strategies that correspond to universally recognized standards for healthy living (e.g. avoiding illicit drugs and excessive consumption of alcohol, getting adequate sleep) [23-25].

Studies of individuals and families with BD suggest that patients and families often vastly overestimate the absolute risks of depression development in first-degree relatives [2,12]. Furthermore, in our previous work involving individuals from families with multiple cases of MDD and BD, we found that concerns about increased genetic risk strongly contributed to a reluctance to have children [9,26]. These data are of concern given that reluctance to have children may in part be likely based on overestimations of risks and suggest that psycho-educational interventions need to address inaccurate risk perceptions. Our prior work also ascertained the information needs of families with multiple members with depression [27]. The majority of people reported wished to obtain information directly from health professionals [27]. However, the need for additional information sources was also identified, and videos and accessing information through the internet were highly rated options.

While high-quality websites on depression are available in Australia (e.g. http://www.beyondblue.org.au, http://www.bluepages.anu.edu.au, http://www.blackdoginstitute.org.au) and internationally, they are not targeted to people with a family history of depression. In this study we will develop a world-first online, evidence-based psycho-educational intervention targeting people at increased risk for depression by virtue of their family history. The intervention will offer 'depression literacy’, including education on prevention and treatment strategies targeted to people with a family history of depression; it will not be designed to provide therapy to prevent or treat depression. The evidence for efficacy of such interventions has been illustrated: several high-quality online interventions that deliver therapy are already available (e.g. MoodGYM) [28]. Evidence is also available that an online intervention focused on depression literacy (e.g. BluePages) is as effective as online CBT (MoodGYM) in reducing the symptoms of depression [29].

The identification of people at increased risk for genetic conditions is particularly challenging because of the unique nature of shared DNA within families, generating implications for whole families rather than merely the individuals involved. Thus, family dynamics and communication patterns can play a major role in determining how information about genetic risk is shared within the family and how individual family members make decisions about, for example, reproduction, early detection and prevention of depression.

Depression with a hereditary component raises several unique issues which call for a sophisticated and rigorously tested approach to patient education. First, information on one’s familial susceptibility to a psychiatric disorder has the potential to impact on the individual’s sense of well-being and integrity and, in the most vulnerable cases, to precipitate the feared condition [30]. Another feature that is likely to compound the delivery of psycho-education is the stigma associated with psychiatric disorders [30,31]. In contrast to individuals with chronic physical conditions, those with psychiatric disorders are amongst the most highly stigmatized groups in society [32,32], regardless of the degree of genetic contribution. In our prior work we found that endorsement of a genetic model for depression was strongly correlated with perceived stigma, which suggests that having a genetic explanation for depression has the potential to further exacerbate the stigma associated with this condition [9]. These data show that a psycho-educational intervention needs to be highly sensitive to the challenges faced by people at increased hereditary risk for depression; our intervention, therefore, aims to provide accurate and realistic information about familial risk without increasing the perceived stigma associated with depression [34]. To address the unique challenges posed by depression with a hereditary component, this project employs a sophisticated analysis of the existing empirical findings on genetic risk for depression and a complex and rigorous methodology.

### Study objectives and hypotheses

This study aims to evaluate the first online psycho-educational intervention targeted to individuals with a family history of depression to provide such individuals in a general practice setting with genetic risk information and evidence-rated information on preventive strategies for depression. The intervention to be tested incorporates a risk assessment tool, delivery of education targeted to people with a family history of depression and feedback to general practitioners (GPs) of individuals’ risk status. It will be evaluated in the GPs setting using a cluster RCT.

The primary aim of the study is to determine whether the intervention, compared to the control condition, leads to both greater intention to adopt, and actual adoption of, risk reduction strategies for depression in people at increased familial risk. The secondary aims are to evaluate whether the intervention will lead to: lower levels of depression symptoms and perceived stigma, and better knowledge about genetic and environmental risk factors for depression and efficacy of risk reduction strategies.

The study will test the following hypotheses: compared to a control group of people receiving brief generic information about depression, people receiving the targeted psycho-educational intervention will be more likely to: (i) intend to adopt, and actually adopt, risk reduction strategies for depression; (ii) have lower levels of depression; (iii) have lower perceived stigma related to depression; and (v) possess better knowledge about risk factors and risk reduction strategies for depression, two weeks and six months post-intervention.

### Design

**Figure 1:**
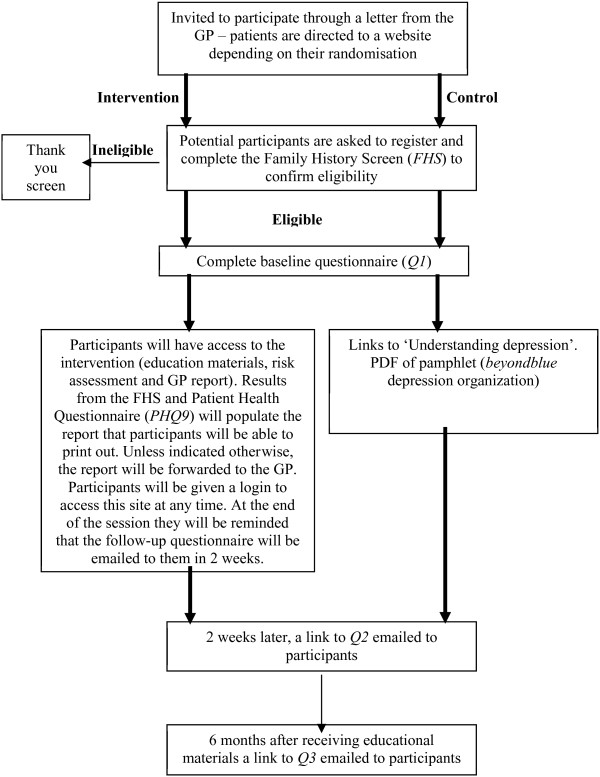
Study design and flow of participants through study.

This cluster RCT will be conducted in a general practice setting in the state of New South Wales, Australia. The study protocol adheres to the CONSORT guidelines [35]. The study design is presented in Figure 1. GP practices will be randomized to provide either access to the online targeted psycho-educational intervention or brief generic information about depression to eligible patients. We decided against an 'attention placebo’ group (e.g. another depression website not targeted to people at high risk for depression) as this would lead to control group participants accessing variable and heterogeneous amounts of education. Instead, participants attending a GP randomized to the control group will receive brief generic information about depression, produced by *beyondblue* (national depression network), i.e. 'Understanding depression’. Randomization by participating GP practice will be undertaken centrally using a computer-based randomization program. Thus the unit of randomization will be the GP practice, not the individual participant. This design will optimize the use of the intervention and minimize contamination between participants attending the same GP; if individual participants rather than GPs were randomized, the approach of the doctor to controls could change due to the doctor’s experience with the tool.

## Methods

### Participants

The study received ethics approval from the institutional review board of the University of New South Wales (approval no 10330) and will be conducted in compliance with the Helsinki Declaration. GP practices will be broadly sampled to ensure heterogeneity and generalizability; both metropolitan and rural GPs practices of varying sizes, including GPs working independently, will be invited to participate. GPs will be identified using an existing GP database in metropolitan Sydney, Australia; GPs will be sent letters of invitation and those returning an expression of interest form will be contacted to arrange a meeting to discuss the study. GP practices will be offered an AUD $400 reimbursement per practice and individual Continuing Medical Education points as an acknowledgement of the time commitments required to participate in the study. The research team will be personalized academic detailing for both groups of GPs provided by in the planning phase of the study, which will include an explanation of the study materials. All GPs will be visited by the researchers to collect baseline demographic and practice details and GPs’ written consent to participate in the trial will be obtained. The research team will continually liaise with and visit participating GPs to ensure adherence to the protocol.

The patient sample will include people with a family history of at least one first-degree relative with MDD or BD who: (i) are able to give informed consent; (ii) are proficient readers in English; (iii) are aged between 18 and 75 years; and are deemed fit by their GP to participate. Patients attending participating GP practices who meet the eligibility criteria will be invited to the study. As many GPs do not elicit and systematically record a family history of depression, patients will be asked to self-identify as having a first-degree degree relative with MDD or BD. Both individuals who have had an episode of MDD or BD, and those who have not, will be eligible to participate.

### Study procedures

Researchers will work with staff at GP practices to generate random lists of up to 500 patients per practice who meet eligibility criteria (i) to (iii) above and have attended the practice in the last two years. GPs will be asked to screen these randomly selected patients and exclude those who would not be suitable (e.g. cognitive impairment, severe illness, patients with a psychiatric disorder that is currently not well controlled); those deemed suitable will be sent a letter of invitation by their GP. Potential participants will be blinded to their intervention assignment in that they will be told that the study is comparing different types of educational interventions. There will be two separate secure study websites, one with the intervention and one with the control condition. The letter of invitation will direct interested individuals to the appropriate study website according to GP randomization. Participants will opt-in and be asked to provide a current e-mail address; participants will then receive a unique username and password that will enable them to access the appropriate website.

Each participant entering the websites (both intervention and control) will complete the *Family History Screen (FHS)*[36] to confirm eligibility (in particular whether they have a first-degree relative with MDD or BD) and provide the basis for feedback to the participants and optionally their GP regarding their family history details (intervention group only), including whether they have had an episode of MDD or BD and brief details regarding their family history. Ineligible individuals will be thanked and asked to discontinue participation, while eligible participants will proceed to complete the baseline questionnaire. The *FHS* is a valid and reliable measure of psychiatric family history and has been shown to have high sensitivity and specificity for depression [36]. It identifies the status of the biological first-degree relatives for assessment (pedigree collection) and screens lifetime history of MDD and/or BD of the participant and the biological relatives identified in the pedigree. The *FHS* begins with a broadly sensitive introductory question about the participant and any first-degree family member to stimulate memory. If the status of any family members is provided in response to the first question, an additional five narrower 'symptom definition’ questions are asked.

### Measures

#### Socio-demographic data

At baseline, data on sex, age, highest level of education achieved, current marital status and country of birth will be collected.

#### Outcome measures to be administered at all time points

##### Patient health questionnaire depression (PHQ9)

The *PHQ9* is a ten-item self-administered validated instrument, which scores each of the Diagnostic and Statistical Manual of Mental Disorders (DSM-IV) criteria for MDD [37]. It can be used both as a diagnostic measure as well as a symptom severity measure and assesses depression symptoms over the past two weeks. It has 4-point Likert type response options ranging from “Not at all” to “Every day”; scores range from 0 to 27, with higher scores indicating higher levels of depression. Depression levels can be categorized as mild (5 to 9 scores), moderate (10 to 14 scores), moderately severe (15 to 19 scores) or severe depression (20 to 27).

##### Perceived devaluation-discrimination

This 12-item scale will be used to assess perceived stigma of depression. It assesses people’s perceptions of what “most other people” believe regarding the stigma associated with depression, a key feature of modified labeling theory according to which perceived devaluation–discrimination should have no impact on social or psychological functioning in people who have never been officially labelled with mental illness [38]. The measure was selected on the basis of its sound theoretical basis and because it has been used mainly among people being treated for mental illness. Items explored beliefs, such as whether a person with bipolar disorder is just as trustworthy as the average citizen, and whether one would willingly accept a person with bipolar disorder as a friend [38]. Four-point Likert-type response scales will be used, ranging from 1 “Strongly disagree” to 4 “Strongly agree” [39], and items will be summed and divided by the total number of items answered, with higher values indicating greater perceived stigma.

##### Knowledge of risk factors for development of depression and risk-reducing strategies

10 items have been purposively designed to assess knowledge of the: (i) genetic and environmental risk factors for depression development and (ii) efficacy of a range of prevention strategies that are covered in the intervention. Item wording is shown in list below. A score of one will be given for each correct answer and a score of zero for an incorrect or “don’t know” response. Total knowledge score will be calculated by summing the correct responses (range 0–10).

##### Perceived susceptibility to major depressive disorder and bipolar disorder

Two items adapted from a previous study [40] will assess: participants’ perceived risk of “developing (or having a recurrence of) MDD” and “developing (or having a recurrence of) BD” compared to an “average person of the same age and gender”, using Likert-scale response options ranging from 1 (“Much lower”) to 5 (“Much higher”).

##### Intention to adopt, and actual adopting of, risk-reducing strategies

Eight items will be used to measure participants’ intention to adopt, and actual adoption of a range of 'risk-reducing strategies’ that were covered in the intervention; only strategies for which at least some evidence of efficacy (prospective data or stronger evidence) is available will be included in this measure. Response options include: “No, and I do not intend to in the next six months” (0), “No, but I intend to in the next six months” (1), “No, but I intend to in the next 30 days (2)”, and “Yes, I have been, but for less than six months” (3), and “Yes, I have been for more than six months” (4). Scores for each item represent the five stages of change: Pre-contemplation (0), Contemplation (1), Preparation (2), Action (3), and Maintenance (4) [41,42]. For analyses, a total mean score will be calculated (range 0 to 4) to assess individuals’ intention and actual adopting of risk-reducing strategies. In addition, for each strategy where participants indicated “Yes”, they will also be asked “how successful they have been” in implementing the strategy. Four-point Likert-type response options will be used, ranging from "Slightly successful" to "Completely successful".

##### Knowledge of risk factors for development of depression and risk-reduction strategies

Environmental factors count for about 40% of developing major depressive disorder. (False)

Genetic factors count for about 80% of developing bipolar disorder. (True)

Having a family history of depression means that a person will definitely get depression. (False)

Chronic stress or having a disability or long-term health condition can increase one’s chances of developing depression. (True)

Being female and drug misuse both can decrease the chance of developing depression. (False)

Parental style does not appear to increase chance of developing depression in people with a family history of depression. (True)

Psychological therapies are very effective in preventing the development of depression. (True)

Regular exercise is definitely not effective in preventing the development of depression. (False)

Lifestyle behaviors such as getting enough sleep, eating a Mediterranean diet, and getting enough vitamin D are definitely not effective in preventing the development of depression. (False)

Having a positive attitude, good social support and greater spiritual well-being may be effective in preventing the development of depression. (True)

##### Intention to adopt, and actual adoption of, risk-reduction strategies

We would like to know whether you would make changes to your lifestyle to reduce your chance of developing depression. Please indicate from the list below how likely you would be to undertake these risk-reducing strategies. Have you...

…used psychological therapy to reduce your chance of developing depression?

…been exercising at a moderate-intensity for 30 minutes of each day to reduce your chance of developing depression?

…been getting adequate amounts of sleep (7-9 hours of sleep each day) to reduce your chance of developing depression?

…been eating a Mediterranean-style diet (rich in vegetables, fruits, nuts, whole grains and fish) to reduce your chance of developing depression?

…been getting the recommended daily intake of vitamin D (either from the spending time in the sun or through food and supplements) to reduce your chance of developing depression?

…been getting good social support to reduce your chance of developing depression?

…been maintaining a positive attitude towards most things to reduce your chance of developing depression?

…joined a religious group or developed your spirituality to reduce your chance of developing depression?

### Intervention

Following completion of the baseline questionnaire, intervention group participants will be directed to the intervention, which is entitled 'Understanding depression that runs in families’, and has three components: (i) feedback of family history assessment and screening test for depression symptoms; (ii) psycho-educational materials, which will be presented in the form of screens containing written information, illustrations and photos; (iii) short video clips, featuring two experts in depression and actors, which will supplement the information covered in the written text; and (iv) an interactive component where participants will be asked to rank the risk-reducing strategies they would like to implement. Upon entry, the results from the family history risk assessment and the screening test for depression (PHQ9) will be fed back to both the participant and, if the participant opts to share the results with their GP, to the GP, in the form of a one page report. Feedback to the GP will via e-mail or fax, according to the GP’s preference, for inclusion in the patient’s file; or the participant may opt to provide the GP with a paper copy of his/her results. The printable or viewable feedback will include details on the participant’s family history as ascertained by the Family History Screen and one of five depression screening feedback reports, with different recommendations depending on the participant’s depression severity category: do nothing, or recommendations regarding follow-up screening, treatment plan, initiation of treatment and/or referral to a mental health specialist [43]. The intervention was developed using an iterative process involving a multidisciplinary team, including one consumer representative. The development and pilot-testing of the intervention will be reported separately in detail. The content and format of the intervention was based on the information preferences expressed in a previous qualitative study of 23 people with a family history of MDD [Quinn V*, Meiser B*, Wilde A, Cousins Z, Barlow-Stewart K, Mitchell P, Schofield P: Preferences regarding targeted education and risk assessment in people with a family history of major depressive disorder *joint first authors, submitted], where most participants reported being interested in accessing depression information via the internet. Also, while we recognize that a web-based tool excludes those without Internet access, it is the best means of reaching the largest numbers of individuals at a very low cost. It can also be readily updated as evidence accumulates in this rapidly developing field of genetics and depression.

The intervention meets the Medical Research Council’s (UK) criteria of a complex intervention (http://www.mrc.ac.uk/complexinterventionsguidance); the Medical Research Council’s conceptual framework guides the development and testing of the intervention. The intervention was developed to be completed in either one only session or several sessions. Some of the intervention content is more relevant to participants who have never had MDD or BD (e.g. the sections about the risk estimates of developing depression based on family history), while other sections may be more relevant to those have had an episode of MDD or BD (e.g. sections on likelihood of recurrence). However, the intervention was designed for participants to navigate website content based on their personal preference. In brief, the psycho-educational intervention includes sections on: genetic and individual and shared environmental factors; differences in genetic information and depression risk; estimating one’s risk of depression development based on family history; risk factors for recurrence of depression; role of environmental factors, including stressful life events, in determining depression risk; strategies to reduce and/or prevent depression development; childbearing decisions; suicide risk; stigma in relation to having a family history of depression; early detection, especially in children and adolescents; and early intervention. The written text of the online intervention is designed to be printable for those who would like a paper version of the tool. The sections on estimating one’s risk of depression development based on family history are presented using pictographs (also called icon arrays or “one hundred people” diagrams) to maximize comprehension [44].

### Control

Following completion of the baseline questionnaire, control group participants will be directed to a PDF of a brief generic pamphlet on depression produced by *beyondblue* (National Depression Network), 'Understanding depression’, which includes information about common symptoms of depression, criteria used to diagnose depression and treatments available. Following completion of the six-month survey, control group participants will be provided with access to intervention.

### Sample size and statistical power

The primary outcome measure will be 'intention to adopt risk-reducing strategies’; on the basis of our quantitative survey of a large nationally representative population sample of 1,046 Australian participants, we expect a mean baseline percentage of 50% of people intending to adopt a range of risk-reducing strategies [45]. Behavioral intentions were chosen as the primary outcome variable to gain an understanding of the issues related to this topic and while behavioral intentions are only an indirect measure of actual behavior, at present they are the best single predictor of future behavior in the psychological literature [46,47].

According to as yet unpublished data from the 2010 National Survey of Mental Health and Wellbeing, at least 20% of Australian residents have at least one first-degree relative with either MDD or BD. Based on similar work involving the testing of a cancer family history assessment tool, it is estimated that approximately 15% of patients will opt-in to an online trial following an invitation letter from their GP [48]. By contacting up to 500 patients for each GP practice, we expect to recruit up to 15 participants per practice.

Our sample size calculations assume an intra-class correlation coefficient (ICC) of 0.06 for patients within the same practice, consistent with ICCs observed for psychological problems in patient encounters in Australian general practice [49]. Assuming an average of twelve participants within each practice, randomizing a total of 20 GP practices will provide 80% power to detect a difference of 22% in the proportion intending to undertake risk-reducing strategies from a control proportion of 50%. This difference, corresponding to a medium effect size [50], is based on the smallest effect that would have clear public health significance and provides a sensitive indicator of clinically meaningful differences.

### Statistical methods

Analysis for the dichotomous primary outcome, intention to adopt risk-reducing strategies, will be performed using the generalized linear mixed model, which extends the standard logistic regression model to allow for clustered data. The continuous outcome of *PHQ9* will be analyzed using the linear mixed model. Adjustment for any potential confounding variables (such as age, sex, type of family history) will be made if there is important baseline imbalance in these variables between intervention groups.

## Discussion

MDD is predicted to contribute the second most disabling largest burden of disease worldwide by 2030 [51]. Given the current climate of cost constraints, a strong need exists to selectively target initiatives to prevent the development of depression in individuals at increased familial risk. The relative risk for depression is substantially increased in close biological relatives of people with MDD and BD. Thus many individuals with a family history of depression are concerned about their personal risk and that of their offspring and report unmet psycho-educational needs on how psychiatric risk can be modified and what steps can be taken to prevent depression.

Currently no research is available on the efficacy of psycho-educational resources specifically aimed at people who are at increased risk of depression due to their family history, despite evidence that such individuals have many unmet information needs. If proven effective, the intervention can be readily adapted to other contexts, for example, as a stand-alone tool or a communication aid to supplement consultations provided by psychiatrists and genetics health professionals to people at increased familial risk for depression. The online psycho-educational intervention will also provide a model for similar interventions aimed at individuals at increased genetic risk for other psychiatric disorders.

An innovative online psycho-educational intervention has been developed, which will be rigorously evaluated to provide individuals at elevated risk in a general practice setting with genetic risk information and evidence-rated information on preventive strategies for depression. A core recommendation of recent governmental policies internationally is the role of primary care in identifying high-risk populations for a wide range of chronic diseases and the promotion of targeted, evidence-based interventions for disease prevention [29]. Primary care is an ideal setting for the identification of people at increased familial risk for psychiatric disorders and a natural setting for implementing preventive strategies. Family history is an important risk factor for many chronic diseases, and family history assessment tools have been developed and evaluated in the general practice setting to identify people with a family history of chronic disease, including breast, ovarian and/or colorectal cancer and/or ischemic heart disease [52]. To our knowledge no tools are currently available for use in the general practice setting to identify people with a family history of psychiatric disorders, including depression, although such tools have been developed for the research setting, e.g. the Family History Screen [36]. Family history assessment tools for other chronic diseases cannot be used to provide a model for interventions for individuals at increased familial risk for psychiatric disorders, including depression, due to the unique nature of these disorders.

The intervention to be tested will provide individuals at increased risk with information on evidence-based strategies for the prevention of depression, thus enabling them to make appropriate lifestyle choices and implement behaviors designed to reduce their risk for depression development. It is anticipated that the novel intervention targeting individuals at increased familial risk will lead to a better understanding of the broad associated genetic risks of depression, increased use of risk-reduction strategies, lower psychological distress and perceived stigma, greater consumer satisfaction, and increased motivation to adopt preventative strategies to reduce the risk for depression.

## Abbreviations

BD: Bipolar disorder; CBT: Cognitive behavioral therapy; FHS: Family history screen; GP: General practitioner; ICC: Intra-class coefficient; MDD: Major depressive disorder; NSW: New South wales; PHQ9: Patient health questionnaire 9; RCT: Randomized controlled trial.

## Competing interests

The authors declare that they have no competing interests.

## Authors’ contributions

BM, PRS, AW, and PBM conceived the study. All authors made substantial contributions to the design of the study, development of the intervention, and/or acquisition of funding. BM wrote the first draft of the manuscript and all co-authors have been involved in reviewing drafts of the manuscript and revising it critically for important intellectual content. MP has a lead role in coordination of the study. All authors have provided their final approval of the current version of the manuscript to be published.

## Pre-publication history

The pre-publication history for this paper can be accessed here:

http://www.biomedcentral.com/1471-244X/13/325/prepub
